# Spatial Patterns of Breast Cancer Risk Associated with Industrial and Environmental Pollutants: A Scoping Review

**DOI:** 10.3390/toxics14020139

**Published:** 2026-01-30

**Authors:** Darashagam Nahal, Abigail Hoffpauir, Kush Kinariwala, Priscilla Tetteh, Francesca Orenge, Anjali Patel, Ashreen Ghalib, Kari Northeim

**Affiliations:** 1 College of Public Health, University of North Texas Health, Fort Worth, TX 76107, USApriscillatetteh@my.unt.edu (P.T.); francescaorenge@my.unthsc.edu (F.O.);; 2 Texas College of Osteopathic Medicine, University of North Texas Health, Fort Worth, TX 76107, USA; laurenhoffpauir@my.unthsc.edu (A.H.); kushkinariwala@my.unthsc.edu (K.K.); anjalipatel@my.unthsc.edu (A.P.)

**Keywords:** breast cancer, environmental pollutants, industrial exposure, geographic analysis, spatial epidemiology, environmental justice, scoping review

## Abstract

This scoping review examined published evidence linking environmental and industrial exposures to breast cancer, synthesizing studies conducted between 2015 and 2025. Using the Arksey and O’Malley framework, 51 peer-reviewed studies were identified and analyzed across five domains: study design, evidence quality, pollutant associations, geographic emphasis, and research gaps. Most studies used retrospective designs, primarily case–control, ecological, cross-sectional, and cohort approaches, which identified associations but could not establish causation. Evidence of quality varied due to heterogeneous environmental modeling methods, exposure to misclassification concerns, and unmeasured confounding, even though 86 percent of studies had sample sizes larger than 1000 cases. Pesticides, polycyclic aromatic hydrocarbons (PAHs), and polychlorinated biphenyls (PCBs) were consistently associated with breast cancer, and nitrogen oxides (NOx), particulate matter (PM), and endocrine-disrupting chemicals (EDCs) also showed frequent significant associations. Research was geographically concentrated in North America and Europe, and few studies examined industrial hotspots or low-income regions. Gaps included the need for stronger epidemiological designs, multipollutant models, standardized exposure metrics, and clearer integration of significant environmental findings into public health protections. Overall, while evidence of pollution-related breast cancer risk continued to accumulate, the precautionary principle remained largely unimplemented. Advancing environmental policy, improving exposure transparency, and incorporating hotspot-based approaches are critical for reducing pollutant burdens and strengthening cancer prevention.

## 1. Introduction

Breast cancer is the most widespread malignant disease and the leading cause of cancer-related deaths among women worldwide [1,2]. According to the World Health Organization [3] (n.d.), more than 2.3 million new cases were reported globally in 2022, resulting in approximately 670,000 deaths. While incidence rates have risen in both developed and developing regions, survival outcomes remain less favorable in underdeveloped areas and among vulnerable women in developed regions [4]. Research over the past few years has identified several genetic and lifestyle risk factors including inherited changes in deoxyribonucleic acid (DNA) repair genes, including breast cancer gene 1 (BRCA1) and breast cancer gene 2 (BRCA2), obesity, alcohol consumption, reproductive history, and hormonal therapy use have been identified as contributing factors to breast cancer development [5,6]. However, growing evidence [7,8,9] links environmental chemical exposures to breast cancer and illustrates that while evidence on specific chemicals, genetic susceptibility, and timing of exposure exists, there remains a fragmented and inconsistent understanding of how industrial environmental exposures collectively influence breast cancer incidence. Industrial pollutants such as heavy metals, dioxins, and endocrine-disrupting chemicals often persist in the environment and affect populations living near industrial areas. Identifying these risks supports stronger environmental regulations and promotes health equity by protecting vulnerable communities from harmful exposures.

Industrialization has transformed ecosystems and human health through the release of a wide array of toxic substances into the environment [10]. The environmental exposure pathway concept illustrates how pollutants from industrial processes such as manufacturing, petrochemical refining, mining, and waste incineration can contaminate air, water, and soil, thereby entering human biological systems through inhalation, ingestion, or dermal absorption [11]. Among these pollutants, several are known or suspected carcinogens and endocrine-disrupting chemicals (EDCs), including benzene, polycyclic aromatic hydrocarbons (PAHs), polychlorinated biphenyls (PCBs), dioxins, and heavy metals such as cadmium and lead [12,13].

Chronic exposure to these carcinogens may influence breast cancer risk through multiple biological mechanisms [14]. EDCs can interfere with hormonal regulation, disrupting estrogen and progesterone pathways that regulate breast tissue development and cell proliferation, while genotoxic agents can induce DNA damage and oxidative stress leading to malignant transformation [15,16]. Nevertheless, establishing a definitive causal relationship between environmental and industrial exposures and breast cancer remains challenging. The disease’s long latency period, combined with varying exposure intensities, complex mixtures of pollutants, and differences in exposure assessment methods, creates uncertainty in existing epidemiological evidence [17,18]. These complexities highlight the need for a comprehensive synthesis of current research to explore environmental and industrial exposures, which may lead to an increased yet often overlooked risk in breast cancer etiology.

Building on the mechanistic understanding that highlights potential carcinogenic pathways, several studies have examined how environmental and industrial exposures vary geographically in relation to breast cancer incidence. Prior studies have reported associations between residential proximity to industrial air emissions or heavy-metal contamination and breast cancer incidence [19,20,21,22]. However, the evidence linking environmental and industrial pollutants to breast cancer remains inconsistent due to variation in exposure assessment methods, heterogeneity in study designs, and limited control for key confounding factors [23,24].

In addition, most spatial and industrial exposure studies examining breast cancer have been conducted in North America and Western Europe [25], while evidence from Africa, Latin America, and much of Asia remains limited. Few population-based studies in these regions have assessed industrial or environmental pollutant exposures in relation to breast cancer, largely due to limited cancer registry coverage and insufficient environmental exposure data [26,27]. This geographic imbalance highlights the fragmented and region-specific nature of existing research and underscores the need for a comprehensive synthesis to map global evidence on how industrial and environmental exposures shape spatial disparities in breast cancer risk.

Although the relationship between environmental exposures and cancer risk has been examined previously, the existing literature remains dispersed across different pollutant classes, geographic contexts, and methodological frameworks. Many studies focus on individual exposures or specific regions, making it difficult to assess broader patterns or compare findings across locations. Few reviews have systematically summarized this literature with attention to how environmental exposures are measured and reported across geographic contexts. This scoping review therefore aims to synthesize recent studies examining industrial and environmental pollutants and breast cancer, while identifying recurring patterns, regional gaps, and methodological limitations that affect interpretation and application of the evidence.

Given the methodological heterogeneity, inconsistency of reported findings and geographic imbalance of existing evidence, a scoping review is the most appropriate approach to synthesize the available evidence with the goal of mapping of the breadth, nature, and methodological diversity of studies spanning epidemiological and environmental science. This approach will identify key exposure types and measurement, study designs, regional patterns, and research gaps, thereby clarifying how industrial and environmental pollutants have been investigated in relation to geographical patterns of breast cancer risk. These insights are essential to inform environmental monitoring, urban planning, and exposure-reduction strategies aimed at cancer prevention. The aim of this review is to comprehensively map and synthesize global evidence on the relationship between environmental and industrial exposures and the geographic patterns of breast cancer incidence. Specifically, it will (1) describe the primary ambient exposures examined, (2) summarize geographic and methodological trends, and (3) identify research gaps and limitations in current study designs. Overall, the review will clarify the current state of knowledge, highlight methodological weaknesses, and propose priorities for future research.

## 2. Materials and Methods

### 2.1. Study Design and Framework

This review followed a scoping review design, which was used to map and summarize existing research on geographic and environmental factors related to breast cancer. The goal was to identify how different studies have examined these relationships and to describe the main patterns, methods, and research gaps in the field.

Studies used a variety of observational designs, including ecological, case–control, cohort, and cross-sectional studies. These studies varied in how exposures and outcomes were measured but were connected by their focus on spatial or environmental patterns and breast cancer. The scoping framework guided the review toward summarizing evidence and highlighting areas that require further investigation.

### 2.2. Research Questions

The review was designed to address five primary questions:

1. What design methodologies are commonly used to examine environmental exposures and breast cancer risk?
2. What is the quality and nature of the evidence linking ambient exposures to breast cancer?
3. What specific environmental exposures are most frequently associated with breast cancer risk?
4. Which geographical locations have been most studied in this context?
5. Where are the research gaps?

### 2.3. Eligibility Criteria

Eligibility criteria for this study were structured using the PICOS (Population, Intervention, Comparator, Outcome, Study Characteristics) framework. (1) Population: included females (sex assigned at birth) of any age from any worldwide location; excluded males, studies without gender stratification, and animal studies; (2) Intervention: included endocrine disruptors, known carcinogens, toxic air pollutants, or ambient exposures; excluded biological factors, lifestyle factors (diet, alcohol), psychosocial stressors, medical treatments, or non-toxic environmental factors (light, noise pollution); (3) Comparator: no specific comparator required; (4) Outcome: included breast cancer, breast malignancy, breast carcinoma, or ductal carcinoma in situ (DCIS); excluded other cancer types, benign breast conditions, non-specific outcomes (all cancers combined), secondary outcomes unrelated to breast cancer (survival rates only), and screening or prevention-only studies; (5) Study Characteristics: included peer-reviewed articles published in English between 2015 and 2025 (with one eligible article published in French) that contained geographical or location-specific details and measured incidence, prevalence, or specific biological markers relevant to breast cancer.

### 2.4. Search Strategy

A comprehensive literature search was conducted across four electronic databases (PubMed, Scopus, Web of Science, and Embase). The strategy combined three core concept groups using Boolean operators: (1) breast cancer terms (e.g., “breast cancer,” “mammary carcinoma,” “breast neoplasm,” “ductal carcinoma,” “lobular carcinoma,” “triple-negative breast cancer,” “HER2-positive breast cancer”); (2) geography and spatial analysis terms (e.g., “geography,” “spatial analysis,” “geospatial analysis,” “spatial distribution,” “spatial epidemiology,” “GIS,” “geographical clustering,” “regional variation”); and (3) environmental exposure terms (e.g., “endocrine disruptor,” “endocrine disrupting chemicals,” “EDCs,” “environmental carcinogens,” “toxic air pollutants,” “ambient exposure,” “chemical exposure,” “chronic exposure”). Wildcard characters (*) were used where appropriate to capture variations in terminology. The search string was adapted for each database to accommodate indexing and syntax differences. Searches were limited to publications from 2015 onward, with no language restrictions. All results were exported into Covidence for deduplication and screening.

### 2.5. Study Selection

All retrieved records were imported into Covidence, where duplicate citations were automatically identified and removed. The study selection process followed PRISMA-ScR guidelines and involved two stages. During title and abstract screening, two independent reviewers screened all records against the predefined eligibility criteria using Covidence’s highlighting feature to identify keywords indicating inclusion or exclusion. Studies marked for inclusion by both reviewers advanced to full-text review. During full-text review, two reviewers independently assessed each article for final eligibility. Disagreements at both screening stages were resolved through discussion, and when consensus could not be reached, a third reviewer resolved the conflict.

Full-text exclusion reasons were customized in Covidence and included the following: (1) not peer reviewed; (2) wrong outcomes; (3) breast cancer not examined as an outcome; (4) wrong intervention; (5) not specific to breast cancer; (6) wrong study type; (7) examined biological or lifestyle factors; and (8) wrong exposure. The number of records identified, screened, excluded, and included at each stage was documented using a PRISMA-ScR flow diagram.

### 2.6. Quality Assessment

Although formal critical appraisal is not mandatory for scoping reviews, an assessment of methodological quality was undertaken to characterize the rigor of the existing evidence. Due to the heterogenous nature of these studies, an appraisal of methodological quality and risk of bias was conducted using a Joanna Briggs Institute (JBI)-aligned, domain-based framework adapted for observational environmental epidemiology studies (Table A1). The framework evaluated study design, population and sampling, exposure measurement, outcome measurement, confounding control, and data quality/bias. Each study was independently reviewed by two authors, and results were reached by consensus.

### 2.7. Data Extraction

Data extraction was completed using a structured form that was developed prior to the review to maintain consistency and accuracy across studies. Two reviewers extracted information independently, then compared results and resolved any differences through discussion. The process focused on capturing key study characteristics and outcomes that aligned with the objectives of the review.

The extraction form included fields for author, publication year, country, and study design, along with details about the study population such as sample size and geographic setting. Information on environmental exposures was collected, including the exposure type, specific pollutants measured, and data sources such as monitoring stations, satellite imagery, or Geospatial Information System-based models. Outcome data were recorded by association and study design. Reported effect estimates, confidence intervals, and *p*-values were included to capture the strength and direction of associations.

Potential confounding variables were noted, and any limitations or biases discussed by study authors were summarized. Extracted data were reviewed for completeness and accuracy before synthesis. All data management, reviewer notes, and consensus tracking were conducted within Covidence.

### 2.8. Data Synthesis

Data from the included studies were organized and analyzed in a spreadsheet using a structured coding matrix. Key information from each study was entered into the matrix, including study design, exposure type, region, and main findings. During the second stage of coding, studies were grouped and compared to identify common patterns in exposures, geographic focus, and reported associations with breast cancer.

Simple frequency counts and summaries were used to show how often certain designs, pollutants, or results appeared across the 51 studies. This approach helped highlight major trends, consistencies, and research gaps without conducting formal meta-analysis.

### 2.9. Ethical Considerations

This review used publicly available data and did not involve human participants; therefore, institutional ethical approval was not required.

### 2.10. Use of AI Tools

Limited assistance from artificial intelligence software tools was utilized during manuscript preparation. Specifically, ChatGPT (GPT-5.1, OpenAI) and Claude (Anthropic) were employed to support minor editing tasks, including grammar checking, sentence restructuring for clarity, and organizational suggestions for section flow. All substantive content, including study selection, data extraction, analysis, interpretation of findings, and scientific conclusions, was developed independently by the research team. AI-generated suggestions were reviewed and modified by the authors to ensure accuracy and alignment with the study’s objectives and findings.

## 3. Results

### 3.1. Selected Study Characteristics

**Figure 1:**
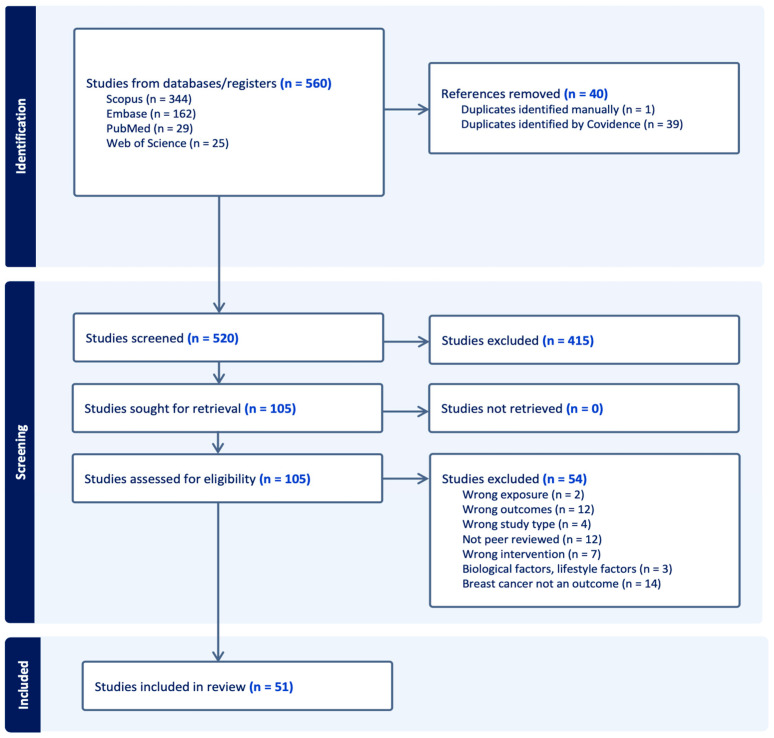
PRISMA Flow Diagram.

In total, 560 studies were identified through the literature search. Following automatic removal of duplicates, 520 studies’ titles and abstracts were screened, with 415 studies excluded. A total of 105 full texts were screened for eligibility based on the inclusion criteria. After final study selection, 51 total studies were included for data extraction and analysis in this review (Figure 1). In total, exclusions were distributed as follows: 12 not peer reviewed, 12 wrong outcomes, 14 breast cancer not examined as an outcome, 7 wrong intervention, 4 wrong study type, 3 examined biological or lifestyle factors, and 2 for wrong exposure. Of the 51 included studies, case–control designs were most common (*n* = 22, 43%). The remaining studies employed ecological (*n* = 10, 20%), cohort (*n* = 9, 18%), cross-sectional (*n* = 6, 12%), and risk assessment or simulation study designs (*n* = 4, 8%) (Table 1). The predominance of retrospective designs reflects the feasibility of linking existing cancer registry data with environmental exposure estimates, though this approach limits temporal inference and causal determination.

### 3.2. Quality and Bias in Results

**Figure 2:**
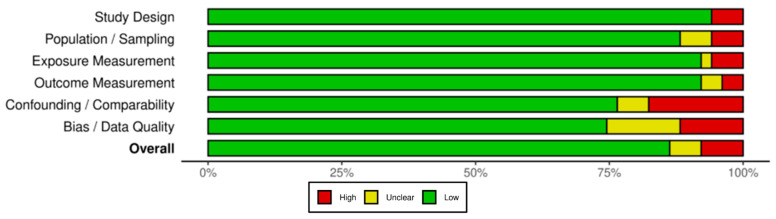
Risk of bias summary plot.

Overall, the quality of the studies included was high, and the risk of bias was low. Most studies clearly stated their design, inclusion, methods, outcomes, confounders, and data. Of the 51 studies included, 44 were classified as having an overall low risk of bias, with only four studies assessed as being high risk, while three studies’ risk of bias remained unclear. A summary of the risk of biased findings is presented in Figure 2.

The primary source of bias was related to data quality and bias control, with some bias in the lack of accounting for or controlling confounding factors. In contrast, the domains of study design, exposure, and outcome measurement exhibited the lowest risk of bias (Figure 2). These findings suggest that while the evidence base is growing, reporting and methodological rigor remain consistent in some domains but lacking in others.

### 3.3. Key Findings and Patterns

This section synthesizes patterns emerging from the reviewed studies, organized into five thematic areas: design methodologies, evidence quality, ambient exposure types, geographical trends, and research gaps. The findings highlight dominant approaches, recurring limitations, and uneven coverage across pollutants and regions.

#### 3.3.1. Design Methodologies

Study designs ranged widely, reflecting differences in available data sources and research goals. Most of the studies employed case–control frameworks (*n* = 22, 43%), often linking environmental exposure indicators to individual cases drawn from cancer registries [19,29,40,67]. Several studies also used prospective cohort designs, allowing temporal assessment of exposures and outcomes [35,50,52,63,69]. Others relied on ecological or spatial analyses, using aggregated exposure measurements or municipality-level indicators [36,47,53,68]. Several studies used model-based or remote-sensing exposure approaches, integrating land-use regression, satellite-based observations, or atmospheric dispersion and chemistry-transport modeling [36,37,61,65,66]. Additional studies described descriptive or trend analyses to examine temporal patterns or spatial clustering of disease [34,48,59]. Finally, several studies combined elements of multiple designs, reflecting hybrid methodological strategies [24,34,39,62,63].

#### 3.3.2. Evidence Quality and Nature of Evidence

Evidence quality evaluation included identification of sample sizes, environmental modeling techniques, stated biases, uncertainties, and overall significance of findings within the included papers. Many studies (*n* = 44, 86%) included more than 1000 breast cancer cases, providing adequate statistical power for detecting associations, while environmental modeling methods varied widely across analytic approaches. Modeled exposure datasets were acknowledged as useful but imperfect, with uncertainties related to spatial resolution, temporal averaging, and emissions inventory accuracy [36,37,61,65,69]. Silva 2019 relied on self-reported datasets and highlighted risks of recall bias, missingness, and exposure misclassification [67]. Investigations using national monitoring networks or remote sensing reported strong spatial coverage but noted inconsistencies across time periods and regions [32,35]. Ecological studies emphasized the difficulty of linking area-level exposures to individual outcomes [36,38,53,60]. Several cohort and case–control studies cited strong internal validity but limited generalizability beyond the populations studied [19,57,58,67].

#### 3.3.3. Ambient Exposure Types Most Frequently Associate with Breast Cancer

Across the 51 studies reviewed, 67% (*n* = 34) reported statistically significant associations between environmental exposures and breast cancer risk (Table 2). An additional 21% (*n* = 11) reported mixed findings, with associations varying by breast cancer subtype (e.g., hormone receptor status), exposure window, pollutant concentration, or population subgroup. The remaining 12% (*n* = 6) reported no significant associations between pollutant exposures and breast cancer outcomes [28,40,42,45,47]. Pesticides showed 100 percent significance (*n* = 5), indicating consistent associations across all studies assessing agricultural, residential, or drift exposures [28,33,41,56,60,67]. PAHs also demonstrated 100 percent significance (*n* = 5), reinforcing strong links between polycyclic aromatic hydrocarbons and breast cancer risk [30,53,68,71]. PCBs showed 100 percent significance as well (*n* = 1), indicating a stable association within this small but consistent evidence base [43]. Traffic-related pollutants, particularly NOx, were significant in 80% (*n* = 4) of studies, further supporting the role of nitrogen dioxide and traffic emissions in breast cancer etiology [31,39,54,66]. Particulate matter showed 70% (*n* = 7) of papers with significance, reflecting consistent associations across PM_2.5_ and PM_10_ exposure windows [32,44,50,52,62,63,73]. Endocrine-disrupting chemicals had 50% of papers with significant findings (*n* = 8), with additional studies reporting mixed or inconclusive findings [58,59,61,69]. Findings for metals were more heterogeneous, with 40% (*n* = 5) showing significant findings, reflecting varying results across cadmium, arsenic, and mixed metal exposures [24,57]. Airborne dioxins papers showed 25% (*n* = 4), with additional studies producing non-significant or mixed findings depending on dose and exposure assessment [64]. Finally, pollutants were categorized as miscellaneous when studies did not specifically assess individual pollutants and instead focused on broad exposure groupings (e.g., “air quality” or “pesticides”), or when studies explicitly evaluated combined pollutant effects. For example, studies examining joint exposure to ethylene oxide and benzene reported higher breast cancer risk [38]. Studies assessing environmental exposures not typically included within major pollutant categories, such as geographic variation in radon exposure, were also classified as miscellaneous [70]. Overall, miscellaneous pollutants were found to be significant in 63% of studies (*n* = 8), emphasizing the importance of integrated exposures when assessing breast cancer risk [19,34,36,38,70]. Studies reporting mixed findings (*n* = 11, 21%) demonstrated the complexity of exposure-disease relationships in environmental breast cancer epidemiology. These studies typically identified significant associations for specific sub analyses—such as particular breast cancer subtypes (ER, HER2 status), exposure timing windows (childhood vs. adulthood), or high-exposure subgroups while finding null or inconsistent results in overall analyses.

#### 3.3.4. Geographical Trends—Urbanicity, Region, Country, Hot Spot

**Figure 3:**
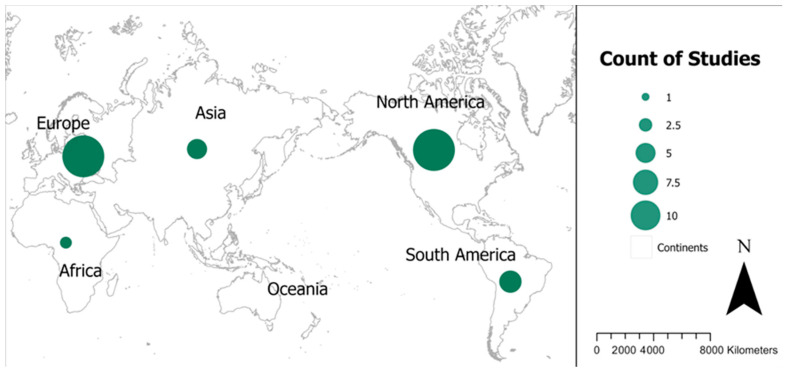
World map displaying included study locations.

Country-level classification revealed geographic patterns across the global research landscape (Figure 3). North American studies (*n* = 18), primarily from the United States and Canada, were well represented [24,35,39,45,46,59,61,72]. European studies (*n* = 18) were similarly prominent and included work from France, Italy, the United Kingdom, Spain, and Denmark [30,36,37,43,44,47,54,60,62,74]. South American studies were fewer in number but contributed important analyses from regions with agricultural expansion and industrial development [41,73]. Asian studies, including those from China, India, and Israel, examined rapid industrialization, dense urbanization, and PM-related exposures [52,58,61]. African representation was limited but included studies from Tunisia and Ethiopia [33,56]. A small number of studies incorporated multicontinental analyses, demonstrating broader international exposure patterns [36,37].

Industrial hotspot classification further differentiated studies based on whether authors explicitly designated regions as high-emission or high-intensity exposure zones. Some studies identifying clear industrial hotspots [46,61,67] while others noted the absence of hotspot conditions [39,40,41,44,74,75]. Collectively, the geographic classifications reveal substantial diversity across the literature, with wide variation in reporting practices and spatial detail. Regional analyses were well represented, while industrial hotspot and geographic specificity remain inconsistently reported across studies.

#### 3.3.5. Stated Gaps

Across the dataset, several patterns emerged in the limitations reported by the studies. A dominant theme was the potential for exposure misclassification, often arising from modeled environmental exposures, reliance on ambient monitoring rather than personal measurements, or incomplete temporal alignment between exposure windows and disease development. Studies describing these challenges emphasized that exposure levels at residential locations may not fully reflect individual exposures due to mobility, commuting patterns, missing historical measurements, or broad geographic proxies [30,35,40,46,60,65,66,67,72,74,76,77].

A second major limitation theme involves incomplete covariates or individual-level data, including the absence of personal risk factors, behavioral exposures, reproductive history, or socioeconomic information. Studies noted that without these data, confounding could not be fully controlled, and risk estimates may be biased. This limitation was particularly common in ecological and registry-based designs, which rely on aggregated data rather than individual participant records [24,34,38,39,42,43,44,46,47]. Finally, some studies reported limitations in outcome measurement, including the inability to distinguish among breast cancer subtypes, limited staging data, or reliance on secondary outcomes such as mortality, density, or survival rather than incidence itself [34,45,57,60,72].

## 4. Discussion

### 4.1. Context and Interpretation

The role of environmental factors in breast cancer etiology has been hypothesized for decades yet remains inadequately integrated into prevention policy despite accumulating evidence. While mechanistic and toxicologic studies demonstrate the carcinogenic and endocrine-disrupting effects of environmental pollutants [78], translating these biological insights into population-level risk assessment and regulatory action remains challenging. This scoping review synthesized the current evidence base linking ambient environmental exposures to breast cancer risk, examining patterns of association, methodological approaches, evidence quality, geographic distribution, and research gaps across 51 studies published between 2015 and 2025.

Our synthesis reveals a paradox: while 67% (*n* = 34) of studies documented statistically significant associations between environmental pollutants and breast cancer risk, the evidence base struggles with limitations that constrain causal inference and policy translation. Research is geographically concentrated in high-income regions (71% from North America and Europe (*n* = 36), relies predominantly on retrospective observational designs (case–control, ecological, cross-sectional), and depends heavily on modeled rather than measured exposure data. Critically, only 10% (*n* = 5) of studies focused on industrial/chemical hotspot populations, who likely face the highest exposure burdens. These methodological and geographic constraints limit our ability to draw definitive causal conclusions or develop targeted interventions for the most vulnerable populations [79].

### 4.2. Methodological Factors (Limitations and Analytical Challenges)

Across the reviewed literature, the majority of studies reported statistically significant positive associations between environmental or industrial exposures and breast cancer incidence. However, the direction and magnitude of associations varied by pollutant class, exposure duration, and regional context. These heterogeneous results likely reflect differences in exposure characterization, population susceptibility, and study design [7,9,80].

The predominance of case–control found during this review is consistent with the historical dominance of retrospective epidemiologic methods in environmental health research. The reliance on case–control design likely reflects data availability and cost-efficiency but also risks items already discussed like exposure misclassification, temporal ambiguity and confounding [81,82,83,84,85].

Temporally, research activity in this area has accelerated in recent years. More than half of the included studies (*n* = 27, 53%) were published between 2021 and 2024, compared to 24 studies (47%) published between 2015 and 2020. This increasing publication trend suggests growing scientific recognition of environmental exposures as potentially modifiable risk factors warranting investigation for breast cancer prevention [86].

Reflecting such limitations of macro-level study design and the commonly cited limitations among the literature reviewed included potential exposure misclassification, unmeasured confounding, and ecological fallacy [87,88]. While most studies adjusted for major individual-level confounders such as age, smoking, occupation, and genetic history, others such as socioeconomic status and race/ethnicity were inconsistently addressed. The absence of standardized approaches to confounder selection and limited data on residential mobility further challenges the internal validity of many studies [86,89].

Interpretation of associations between environmental and industrial exposures and breast cancer risk is further limited by inconsistent adjustment for established risk factors across studies. Many studies included in this review adjusted for only a small number of individual-level variables, while others relied on area-level measures or did not report detailed covariate information. As a result, residual confounding by known breast cancer risk factors such as body mass index, reproductive history, and hormonal factors cannot be excluded. This limitation is particularly relevant for studies of endocrine-disrupting chemicals, given their potential to act through hormonal pathways that may overlap with metabolic and reproductive processes. Few studies in this review evaluated these factors jointly or examined whether associations varied across subgroups, which may contribute to null, inverse, or heterogenous findings in the literature.

Importantly, the focus on evaluation of pesticides and endocrine-disrupting chemicals and particulate matter reflects both their established biological plausibility and the relative availability of monitoring data [90]. These patterns may suggest that research attention has concentrated on well-characterized pollutants rather than the full spectrum of relevant exposures [91]. Although these pollutants may be better-characterized, emerging interest in mixed industrial exposures suggests a shifting research focus toward complex mixtures [91], yet consensus is still lacking their singular influences.

To detect pollutants, many studies relied on modeled or secondary exposure data sources, such as land-use regression (LUR) or ground monitoring, with only a small subset using direct or biomonitoring-based exposure assessment. Although LUR models have developed substantially over the past 2 decades and have a pivotal role in evaluating long- and short-term exposures [92], their assumptions and resolutions vary widely across settings, complicating cross-study comparability [80]. Dependence on modeled exposures, coupled with limited individual pathology, may obscure true dose–response patterns [93,94]. This methodological gap raises concerns about exposure misclassification and highlights the need for temporal and spatial resolution in future studies [95,96].

Analytically, integrating spatial and temporal modeling could improve precision, yet substantial variability persists in how exposures are defined, scaled, and temporally aligned with outcome data [97]. Of interest, several studies showed no association or significance between environmental pollutants and breast cancer when geographical information systems (GIS) spatial analysis was used alone. While GIS has added significant value to the world of environmental health research [98], it may be more suitable in conjunction with other methods to assess complex relationships between the combination of environmental pollutants and breast cancer. Taken together, the reviewed evidence brings to light a paradox in environmental epidemiology: as analytic sophistication increases, comparability across studies often decreases [99].

### 4.3. Geographic Disparities and Environmental Justice

Beyond methodological constraints, the distribution of the evidence itself is geographically skewed, raising issues of representativeness, and global generalizability. With many studies conducted in North America and Europe, the concentration in high-resource settings limits the external validity of findings and overlooks environmental exposures unique to industrializing settings [100]. Emerging work from Asia and South America contributes to valuable diversity, but studies from Africa and other low-income regions remain sparse. The absence of exposure monitoring infrastructure in low-income regions could perpetuate environmental inequity. This pattern mirrors broader inequalities in health research capacity, which in turn results in underserved populations bearing disproportionate exposure burdens without epidemiologic oversight needed to inform prevention.

### 4.4. Future Directions

The review underscores several priorities for strengthening epidemiologic evidence on environmental exposures and breast cancer risk. Firstly, advancing study design remains critical. The limitations in study design are reflected in the fact that most studies were retrospective in nature, including case–control and retrospective cohort approaches, with only a small number using prospective designs. While prospective studies cannot establish causality, they can establish temporality, clarify the sequence of exposure to outcome, and provide insight into the natural history of disease.

Improving exposure characterization is another essential step. Incorporating high-resolution exposure metrics, standardized pollutant groupings, and approaches capable of addressing multiple pollutants simultaneously will enhance comparability across studies. Integrating geographic information systems (GIS), land-use regression, and satellite-based remote sensing can provide more spatially refined and temporally resolved exposure estimates [95,96].

The current inconsistencies that exist among the literature, including variable exposure definitions and measurement metrics, confounder adjustments, and temporal alignment, hinder meta-analysis and adequate estimations of global risk burden. Emerging interest in prospective studies exploring combinations of environmental pollutants and breast cancer incidence are more closely aligned with real-world conditions of human exposure [101,102] and utilizing these methodologies going forward would strengthen temporal inference. The current variability and heterogeneity of cancer risk association studies reflect the difficulty of identifying the cause and effect of a longitudinal and biologically complex process at a geographically large scale.

Finally, expanding these implementations equitably into under-represented areas and vulnerable populations is critical to environmental justice [103]. Collectively, these efforts may facilitate the transition from correlation to causal inference and from hypothesis to actionable evidence.

## 5. Policy and Implications

The findings from this review point to a clear need for stronger policies that focus on prevention, even when full causal proof has not yet been established. Many studies indicate meaningful health impacts from pollutant exposure, especially endocrine-disrupting chemicals and heavy metals. A precautionary approach is warranted, since waiting for definitive causal evidence may allow avoidable harm. Strengthening air toxics standards, improving emissions reporting, and expanding monitoring of high-risk pollutants would better align environmental regulation with current scientific evidence [104,105].

### 5.1. Research Funding and Infrastructure

More investment is needed to support research that can clarify exposure pathways and strengthen causal assessment. Longitudinal and nested designs, biomonitoring, spatial exposure modeling, and multi-sensor data collection are essential for improving exposure precision and identifying sensitive life stages. These studies are resource-intensive but foundational for prevention. As an example, federal initiatives such as the NIH’s Environmental influences on Child Health Outcomes (ECHO) Program demonstrate how continuous investment in longitudinal cohorts, biomonitoring, and harmonized exposure assessment can strengthen causal interference and improve understanding of environmental influence on health and disease throughout the life course [106,107]. Expanding funding for long-term cohorts, environmental sensor networks, and community-based data collection may improve understanding of environmental burdens and guide policies that can prevent disease more effectively.

### 5.2. Advancing Health Equity and Public Health Prevention

Policy and research efforts should place health equity at the center of environmental health action. Increasing monitoring in underserved communities, supporting community-driven prevention programs, and investing in public health education can reduce disparities in exposure and disease risk [108]. Equity-focused policies such as the European Union’s Zero Pollution Action Plan 2030, whose implementation includes explicit attention to reducing social inequalities in pollutant exposures and ensuring that pollution control measures benefit disadvantaged and vulnerable populations [109,110]. Integrating environmental justice principles into this framework aims to mitigate unequal exposure burdens and align regulatory action with public health equity. Clear communication about environmental hazards, mitigation strategies, and early detection can empower affected communities. Integrating these efforts into existing public health systems, including health departments, primary care, and schools, can ensure that prevention strategies reach populations that need them most.

## 6. Conclusions

This scoping review synthesizes evidence from 51 studies published between 2015 and 2025, which revealed that 67% of studies identified statistically significant associations between environmental pollutants and breast cancer incidence, with particularly strong evidence for pesticides, PAH, PCB, NOx, and PM.

Despite this accumulating evidence, critical limitations constrain translation into public health prevention. The evidence base is geographically skewed towards North America and Europe, leaving substantial gaps in rapidly industrializing regions of Asia, Africa, and Latin America, where environmental burdens may be high. Methodologically, the predominance of retrospective design limits causal inference, while reliance on modeled exposure data introduces measurement error and potential misclassification.

Future research must address current limitations through prospective cohort studies with repeated biomonitoring across the life course, standardized exposure metrics enabling meta-analysis, multipollutant mixture models reflecting real-world exposures, and targeted investigation of industrial hotspot populations and underserved communities. Methodological concurrence is essential to enable pooled analyses and global risk estimation.

The disconnect between scientific evidence and regulatory response reflects a challenge in implementing the precautionary principle in environmental health policy. While many studies demonstrate that significant associations and biological mechanisms are well-established, the burden of proof needs to shift toward prevention rather than demanding absolute causal certainty before action. Strengthening air quality standards to account for cancer risk, expanding emissions monitoring in residential areas, integrating environmental exposure data into cancer surveillance systems, and enforcing protective buffer zones near industrial facilities represent evidence-based interventions that could reduce population exposure burdens.

## Figures and Tables

**Table 1:** Study Selection and Characteristics.

Author, Year	Study Objective	Study Design	Study Population	Exposure Type (Data Source)	Geographic Distribution	Results/Summary
Amadou et al., 2020 [28]	Assess chronic long-term exposure to cadmium air pollution and breast cancer risk in the French E3N cohort	Case–control study	9058 women total: 4529 invasive breast cancer cases and 4529 matched controls (nested within the E3N cohort).	Airborne cadmium (Exposure data from national emission inventories (OMINEA, EMEP), industrial databases, and Geographic Information Systems (GIS) geocoded residential histories)	E3N cohort France 1990–2008. Industrial hotspot proximity not assessed	No significant association between airborne cadmium and overall breast cancer risk
Amadou 2020 Erratum [28]	Assess chronic long-term exposure to cadmium air pollution and breast cancer risk in the French E3N cohort	Case–control study	4401 cases and 4401 matched controls	Atmospheric exposure (GIS)	E3N cohort France 1990–2008 Industrial hotspot not assessed	No significant association between airborne cadmium and overall breast cancer risk
Amadou et al., 2021 [29]	Examine exposure to airborne cadmium and breast cancer stage/grade/histology at diagnosis	Case–Control study	3924	Cadmium (GIS)	France	No relationship between cadmium exposure and stage or grade of breast cancer. Positive association between cadmium and risk of invasive tubular carcinoma
Amadou et al., 2021 [30]	Investigate risk of breast cancer associated with long-term exposure to benzo[a]pyrene (BaP) air pollution: Evidence from the French E3N cohort study	Case–control study	5222 cases + 5222 controls	Air pollution (CHIMERE transport model)	France—wide cohort; residential addresses all over metropolitan France (except for Corsica). Industrial hotspot not assessed	Cumulative airborne BaP (benzopyrene) exposure was significantly associated with the overall risk for breast cancer
Amadou et al., 2023 [31]	Assess long-term exposure to nitrogen dioxide air pollution and breast cancer risk within the French E3N cohort study	Case–control study	5222 cases and 5222 matched controls	Air toxics (Annual means concentrations of Nitrogen Dioxide (NO_2_) at participants’ residential addresses)	Metropolitan France. Industrial hotspots reflected high NO_2_ in urban and traffic-dense areas.	Exposure was associated with an increased risk of breast cancer
Arif et al., 2024 [32]	Meta-analysis of the carcinogenic effects of particulate matter and polycyclic aromatic hydrocarbons.	Meta-analysis	24 breast cancer incidence studies	Air pollution (data extracted from literature that was analyzed)	Urban with general outdoor urban air pollution	Increased breast cancer mortality with PM2.
Arrebola et al., 2015 [33]	Investigate association between serum concentrations of compounds with xenoestrogenic potential and risk of breast cancer	Case–control study	69 cases and 56 controls	Organochlorine pesticides and PCBs (human serum levels)	2 Main Specialist Cancer Centers Tunis Metropolitan Area	Potential association between exposure to at least one organochlorine pesticide and breast cancer risk
Calaf et al., 2020 [14]	Analyze EDCs in relation to breast cancer to create model for future analysis of EDCs	Review	N/A	EDCs (literature)	Chile and USA	BPA, Dichlorodiphenyltrichloroethane (DDT)/DDE, and PCBs alter proliferation, are genotoxic, immunosuppressive, induce epigenetic alterations and oxidative stress; DDT and PCBs induce chronic inflammation; BPA also alters DNA repair/genomic instability; PCBs can be electrophilic or metabolically activated. Each suggest breast cancer susceptibility
Carreras et al., 2020 [34]	Investigate burden of disease from breast cancer attributable to smoking and second-hand smoke exposure in Europe	Comparative risk assessment	82,239 Disability-Adjusted Life Years (DALYs) and 3354 deaths from breast cancer	Tobacco smoke and exposure (Eurobarometer Survey)	European Union	Highest burden due to smoking and smoke exposure was estimated in Denmark, Malta, Croatia, Hungary, and United Kingdom
Carroll et al., 2023 [35]	Investigate whether environmental exposures or neighborhood socioeconomic status explain geographic pattern of breast cancer incidence in the U.S.	Prospective cohort study	44,707	NOx, fine particulate matter 2.5 μm (PM_2.5_), (Light at Night (LAN), Ambient noise, Ultraviolet (UV) Radiation, Greenspace (accelerated failure time models with spatial random effect term)	United States	Suggests role of environmental exposures in breast cancer incidence and geographical-based risk factors vary according to breast cancer subtype
Cazzolla Gatti et al., 2023 [36]	Investigate possible significant correlations between spatial distribution of different sources of pollution and cancer mortality	Retrospective ecological study	Italian population (20 regions and 127 provinces)	Air quality, traffic emissions, pesticides, radiofrequency emitters (Regional environmental agencies and ISTAT and Air Quality Index)	Italy (rural, urban, and industrial zones)	Breast cancer mortality showed a stronger link with urban and industrial areas (pollution explained 50% of variation)
Cazzolla Gatti, 2021 [37]	Synthesize and review state of knowledge of links between cancer and environmental pollution	Review	N/A	Pesticides, heavy metals, solvents, PAHs, PCBs, dioxins, asbestos, radon, nitrates, EMF (literature)	Global (emphasis on industrial hotspots)	Environmental triggers may explain increases in breast cancer incidence beyond detection or lifestyle changes
Chen, 2018 [38]	Examine associations between incidence rate of invasive breast cancer and socioeconomic characteristic and environmental risks over time in Illinois	Retrospective ecological study	>8000 cases/year (Illinois State Cancer Registry)	Air toxics (Environmental Protection Agency (EPA) National Air Toxics Assessment)	Illinois	Ethylene oxide and benzene were linked to higher rates. Socioeconomic factors explained most of differences
Cohen et al., 2018 [39]	Assess chronic exposure to traffic-related air pollution and cancer incidence among patients undergoing percutaneous coronary interventions	Retrospective cohort study	9816 participants	Traffic-related air pollution (Land Use Regression (LUR) model, national coverage, 50-m spatial resolution)	Nationwide Israel. Industrial hotspot proximity not assessed	Breast cancer incidence was significantly associated with long-term exposure to traffic-related air pollution.
Coudon, et al., 2020 [40]	Study association between environmental exposure to dietary and airborne dioxins and breast cancer risk	Case–control study	Dietary exposure: 63,830 women (3465 cases); Airborne exposure: 4529 cases and 4529 controls	Airborne dioxins and dietary dioxins (GIS)	France	No statistically significant association observed for dietary dioxin exposure; Rhône-Alpes regional analysis showed no significant association for airborne exposure; National airborne exposure analysis ongoing
Da Silva et al., 2024 [41]	Investigate relationship between mortality from breast cancer and use of pesticides	Retrospective ecological study	118 municipalities	Pesticides known to be EDCs (state pesticide registry)	Mesoregion of Santa Catarina—Brazil (Rural)	Municipalities with higher pesticide use had higher breast cancer mortality after 15 years of exposure
Danjou et al., 2019 [42]	Estimate breast cancer risk associated with airborne dioxin exposure	Case–control study	429 Cases, 716 Controls	Airborne dioxins (GIS)	Entire region—not restricted to industrial hotspot	No clear association between dioxin exposure and breast cancer. Possible non-linear relationship between dioxin exposure and breast cancer
Deygas et al., 2021 [43]	Estimate association between cumulative atmospheric exposure to total PCBs exposure	Case–control study	5222 breast cancer cases and 5222 controls	PCB (deterministic chemistry-transport model—CHIMERE and geocoded residential history)	France	Cumulative PCB153 exposure was tied to higher breast cancer risk
Duboeuf et al., 2024 [44]	Investigate association between PM_2.5_/(fine particulate matter 10 μm (PM_10)_ and NO_2_ atmospheric concentrations at women’s residential/workplace locations and breast cancer risk	Case–control study	2419 cases and 2984 controls	PM_2.5_/PM_10_ and NO_2_ (LUR model)	France	Increased breast cancer risk observed for 10 μg/m^3^ increase in average PM_2.5_/PM_10_ and NO_2_ concentration estimates. PM_2.5_/PM_10_ and NO_2_ residential concentrations strongly correlated with workplace concentrations allowing residential data to serve as proxy for overall exposure
DuPre et al., 2019 [45]	Investigate particulate matter and traffic-related exposures in relation to breast cancer survival	Longitudinal study	Total sample size = 8936 women with stage I-III breast cancer	Air pollution (spatiotemporal models)	Nurses who resided in 11 states unknown whether it was urban or rural. Industrial hotspot proximity not assessed	PM was not associated with breast cancer
Garcia et al., 2015 [46]	Examine relationships between breast cancer incidence and modeled concentrations of air pollutants shown to be mammary gland carcinogens	Prospective cohort study	5676	Air pollutants shown to be mammary gland carcinogens	California	Elevated risk of breast cancer may be associated with some compounds for certain subgroups of interest
García-Pérez et al., 2016 [47]	Examine if excess breast and prostate cancer mortality among population residing near Spanish industries	Retrospective ecological study	57,830 deaths from breast cancer in 8098 Spanish towns between 1997 and 2006	Industrial pollution (Spanish Environmental Ministry and European Pollutant Release and Transfer register (PRTR) Registry)	Spain Industrial Sites	Residing in the vicinity of pollutant industries as a whole is not a risk factor for breast and prostate cancer mortality
García-Pérez et al., 2018 [19]	Assess relationships between risk of breast cancer and residential proximity to industries	Case–control study	1738 breast cancer cases and 1910 controls	EDCs (Euclidean distance and European Pollutant Release and Transfer Register)	Spain (10 provinces)	Woman living within 3 km of any industrial site had higher breast cancer risk
Gearhart-Serna et al., 2020 [48]	Investigate environmental quality and invasive breast cancer relationship	Case–control study	Women diagnosed with breast cancer in North Carolina from 2009 to 2014	Air, land, water pollutants (Environmental Quality Index (EQI))	Statewide in North Carolina. Covered both urban and rural counties. Industrial hot spots not assessed	Overall environmental quality was not strongly linked with invasive breast cancer. But poor land quality stood out. Women in counties with the worst land quality were more likely to have invasive breast cancer compared to carcinoma in situ, especially in rural areas.
He et al., 2016 [49]	Provide insight into evidence for risk of breast cancer with exposure to environmental estrogen-like chemicals	Review	N/A	EDCs (literature)	Global	Exposure to environmental estrogen-like chemicals may increase breast cancer risk
Hvidtfeldt et al., 2023 [50]	Investigate breast Cancer Incidence in Relation to Long-Term Low-Level Exposure to Air Pollution in the ELAPSE Pooled Cohort	Prospective cohort study	Total sample size: 199,719 women; 9659 incident breast cancer cases.	Air Pollution: PM_2.5_, NO (European monitoring stations (AirBase, ESCAPE), satellite images, and computer models that combined land use and road data.)	Predominantly urban and peri-urban settings in Europe. not specifically industrial hotspots	Found a higher risk of breast cancer incidence in relation to higher exposure
Ilozumba et al., 2022 [51]	Urinary Concentrations of Triclosan, Bisphenol A (BPA), and Brominated Flame Retardants and the Association of Triclosan with Demographic Characteristics and Body Fatness among Women with Newly Diagnosed Breast Cancer	Observational, cross-sectional study	302 women with newly diagnosed stage 0 breast cancer.	Endocrine-disrupting chemicals (urine samples)	United States, multi-center hospital-based study. Industrial hotspot not assessed	Obese women had significantly lower urinary triclosan compared with normal-weight women, especially postmenopausal women. BPA and brominated flame retardants were rarely detected.
Kayyal-Tarabeia et al., 2024 [52]	Investigate air pollution and bladder, breast and prostate cancer incidence	Prospective cohort study	Nationwide cohort of 918,046 adults in Israel.	Air pollution: PM_2.5_ (satellite-based Aerosol Optical Depth (AOD) modeling calibrated to monitors)	Nationwide Israel. Both urban and rural areas were included. No industrial hotspot(s) were identified.	Per one IQR increase in PM_2.5_ (2.11 µg/m^3^), breast cancer risk increased: HR 1.50 (95% CI 1.42–1.58) in single-pollutant models.
Large & Wei, 2017 [53]	Assess role of exposure to ambient air pollution in geographical variation in breast cancer incidence	Retrospective ecological study	18 regions	PAHs (EPA national Emissions Inventory)	Northeastern v. Southeaster U.S. regions	Positive links between PAH emissions and breast cancer
Le Provost et al., 2024 [54]	Characterize association between residential exposure to PM_2.5_ and NO_2_ and breast cancer	Case–control study	465 cases and 242 controls	PM_2.5_ and NO_2_ (Spatiotemporal pollution models based on regional air monitoring)	Ontario, Canada	Recent exposure to NO_2_ was significantly associated with increased risk of early onset breast cancer
Liu et al., 2021 [55]	Explore the association of BPA and phthalates with risk of breast cancer	Meta-Analysis of 9 Case–Control Studies	7820 breast cancer cases and controls	Endocrine-disrupting chemicals (urine samples)	America (6 studies), Alaska Native (1 study), North Mexico (1 study), and Poland (1 study). Industrial hotspots not assessed	Phthalate metabolites MBzP and MiBP were passively associated with breast cancer, whereas no associations were found between BPA, MEP, MEHHP, MEHP, MEOHP, MCPP, and MBP and breast cancer.
Mekonen et al., 2021 [56]	Assess exposure to organochlorine pesticides as a predictor to breast cancer	Case–control study	50 cases and 50 controls	Organochlorine pesticides (blood samples)	Oncology unit in Ethiopia	Organochlorines are a risk factor for breast cancer in Ethiopia
Michel-Ramirez et al., 2020 [57]	Assess Yes-Associated Protein (YAP) gene polymorphisms and arsenic interaction in Mexican women with breast cancer	Cross-sectional	77 women with breast cancer and 105 controls with benign breast biopsies.	Heavy metals (Urinary arsenic speciation)	Residents of Comarca Lagunera Region (north-central region of Mexico). High arsenic tap water levels have been detected at this region	Positive and significant associations were found between breast cancer and smoking, type of drinking water, As, As, and iAs, whereas a negative and significant association was found with first methylation
Mukherjee Das et al., 2022 [58]	Assess urinary concentration of endocrine-disrupting phthalates and breast cancer risk in Indian women	Case–control study	171 women total: 90 invasive breast cancer cases and 81 controls recruited at AIIMS, New Delhi.	Endocrine-disrupting chemicals (urine samples)	Both urban and rural populations. Not designed around an industrial hotspot, though Delhi is a highly polluted city with known phthalate exposure sources.	Higher urinary levels of certain phthalates were linked to increased breast cancer risk.
Omoike et al., 2021 [59]	Examine association between (Perfluorinated chemicals (PFCs) and a group of estrogen related cancers	Cross-sectional	11,631 adults	EDC (serum biomarkers)	United States	PFC exposure was linked to higher odds of breast and ovarian cancer
Panis et al., 2022 [60]	Evaluate evidence regarding pesticide contamination in drinking water of municipalities in the state of Parana	Retrospective ecological study	5.5 million people (127 municipalities)	Pesticides (SISAGUA Report of National Program for Monitoring Quality of Water for Human Consumption)	Parana, Brazil	Municipality-level estimated cancer cases from contaminated water strongly correlated with recorded breast cancer cases
Peng et al., 2023 [61]	Identification of breast cancer and associated ovarian/uterus cancer risk components in source waters from high incidence area in Pearl River Basin, China	Cross-sectional & observational study	3 Study regions (populations of 602,000; 375,000; 526,000)	EDCs, Heavy metals, Nitrates (long-term & short-term monitoring programs, and additional reported sampling results)	Pearl-River Basin, China (Rural)	Contaminant levels correlate with higher regional breast cancer incidence
Poulsen et al., 2023 [62]	Examine air pollution with NO_2_, PM_2.5_, and elemental carbon (EC) in relation to risk of breast cancer	Case–control study	French E3N cohort including 5222 breast 55,745 breast cancer cases and 55,745 individually matched controls	Air toxics (modeled using DEHM/UBM/AirGIS system)	Nationwide Denmark. High industrial densities/hotspots identified and analyzed.	Long-term residential PM_2.5_ exposure was consistently associated with increased risk of breast cancer.
Prada et al., 2021 [63]	Long-term PM_2.5_ exposure before diagnosis is associated with worse outcome in breast cancer	Prospective cohort study	151 women with breast cancer treated at Mexico’s National Cancer Institute	Air pollution: PM_2.5_ (AOD model)	Mexico City (urban). Industrial hotspot not assessed	Among women already diagnosed, higher long-term PM_2.5_ (1-year pre-diagnosis) was linked to larger tumors at diagnosis
Praud et al., 2025 [64]	Investigate association between long-term exposure to airborne dioxins and breast cancer	Case–control study	5222 breast cancer cases and 5222 matched controls	Airborne dioxins (GIS)	France	Increased risk of breast cancer associated with long-term residential exposure to dioxins
Shekarrizfard et al., 2015 [65]	Investigate the role of transportation models in epidemiologic studies of traffic related air pollution and health effects	Case–control study	377 postmenopausal breast cancer cases and 415 controls	Traffic-related air pollution (LUR model, national coverage, 50-m spatial resolution)	Dense urban center with high traffic and industrial emissions	A comparison of odds ratios (ORs) obtained from NO_2_ and NOx used in two case–control studies of breast and prostate cancer, showed that the differences between the ORs associated with NO_2_ exposure vs. NOx exposure differed by 5.2–8.8%
Shekarrizfard et al., 2018 [66]	Compare distribution of spatial estimates of NOx and estimates of risk of breast/prostate cancer computed from transportation model and LUR model	Case–control study	792 breast cancer and 1722 prostate cancer participants	NOx (LUR and Air Pollution Dispersion Models)	Montreal, Quebec, Canada (Urban)	Higher long-term NOx exposure was linked to increased breast cancer risk. Stronger associations were seen with land-use regression models.
Silva et al., 2019 [67]	Investigate environmental exposure to pesticides and breast cancer in a region of intensive agribusiness activity in Brazil	Case–control study	351 women (85 cases and 266 controls)	Pesticides (self-reported questionnaires)	Urban (located in a region of heavy agricultural activity and high pesticide use)	In the final model, living near cropland with pesticides (OR: 2.37; CI: 95% 1.7–3.16) and women aged over 50 years who experienced early menarche (OR: 2.08; CI: 95% 1.06–4.12) had a higher risk of developing breast cancer compared to control subjects.
Stults et al., 2018 [68]	Examine ambient air emissions of polycyclic aromatic hydrocarbons and female breast cancer incidence in US.	Ecological study	Ecological study across 194 US counties within SEER 9 regions	Air pollution (2008 US EPA National Emissions Inventory)	SEER regions across the US and industrial hotspots were assessed.	Breast cancer incidence was consistently higher in metropolitan/industrialized regions compared to rural/micropolitan areas. Traffic emissions appeared to be the strongest predictor of breast cancer incidence.
Tang et al., 2024 [69]	Investigate exposure to di-2-ethylhexyl phthalate (DEHP) and breast neoplasm incidence	Prospective cohort study	273,295 women.	Water pollution: Phthalates (DEHP levels interpolated to residential addresses)	United Kingdom, nationwide cohort. Urban vs. rural not explicitly stratified. No industrial hotspot(s) were identified.	Long-term average DEHP exposure in drinking water was significantly associated with increased risk of breast neoplasms
Vieira et al., 2019 [70]	Examine contribution of socioeconomic and environmental factors to geographic disparities in breast cancer risk in the Nurses’ Health Study II	Case–control study	3478 breast cancer cases; 24,519 control women	Radon (Satellite data for LAN and the Lawrence Berkeley National Laboratory U.S. radon model for radon estimates)	48 U.S. states; mainly urban and suburban areas. Elevated risk areas identified in Iowa, Ohio, southern New England, and the New York region (areas of higher population and industrial activity).	Breast cancer risk varied geographically
Waddingham et al., 2024 [71]	Characterize relationship between ambient PAH exposure and early-onset breast cancer risk	Case–control study	435 cases and 222 controls	PAHs (Global Environmental Multiscale Modeling Air Quality and Chemistry)	Ontario, Canada	Women with higher ambient PAH exposure (fluoranthene) had significantly higher odds of early-onset breast cancer
White et al., 2019 [24]	Examine airborne metals and polycyclic aromatic hydrocarbons in relation to mammographic breast density	Cross-sectional study	222,581 women	Air pollution (EPA and National Air Toxics Assessment (NATA))	Urban and suburban regions across five U.S. states. Industrial hotspot proximity not assessed	Women living in areas with higher levels of lead, cobalt, manganese, nickel, arsenic, or PAHs had higher odds of dense breasts
Yaghjyan et al., 2017 [72]	Association between air pollution and mammographic breast density in the Breast Cancer Surveillance Consortium	Cross-sectional study	279,967 women	PM_2.5_ and O_3_ (US Environmental Protection Agency hierarchical Bayesian Model)	United States	Exposures to PM_2.5_ and O_3_ may in part explain geographical variation in mammographic density
Yu et al., 2022 [73]	Investigate associations between long-term exposure to PM_2.5_ and site-specific cancer mortality: A nationwide study in Brazil between 2010 and 2018	Ecological	1,768,668 adult cancer deaths (830,468 women) from a population of ~208 million.	Air pollution: PM_2.5_ (GEOS-Chem CTM, calibrated with MODIS, MISR, SeaWiFS satellite AOD + ground monitors,)	Nationwide, across 5565 municipalities in Brazil (urban + rural included). Industrial hotspot not assessed	Exposure was significantly associated with higher mortality from total and multiple site-specific cancers, including breast cancer.
Zhai et al., 2022 [74]	Analyze bias induced by left truncation in estimating breast cancer risk associated with exposure to airborne dioxins	Simulation (500 random case–control studies)	200,000 virtual women cohort	Airborne dioxins (GIS)	Simulation based on French National E3N cohort	Exposures before enrollment are ignored, estimated breast cancer risks from dioxins are overstated compared with models that include lifetime exposure

**Table 2:** Study Significance Findings from Scoping Review.

Pollutant Category	Total Studies	Significant	Not Significant	Mixed/Inconclusive	Significant Study Citations
Airborne Dioxins	4	1	2	1	Praud 2025 [64]
PCBs	1	1	0	0	Deygas 2021 [43]
NOx/NO_2_	5	4	0	1	Amadou 2023; Cohen 2018; Le Provost 2024; Shekarrizfard 2018 [31,39,54,66]
Metals	5	2	2	1	White 2019; Michel-Ramirez 2020 [24,57]
Pesticides	5	5	0	0	Arrebola 2015; daSilva 2024; Mekonen 2021; Panis 2022; Silva 2019 [33,41,56,60,67]
PAHs	5	5	0	0	Amadou 2021; Large 2017; Stults 2018; Waddingham 2024 [30,53,68,71]
Particulate Matter (PM)	10	7	1	2	Arif 2024; Duboeuf 2024; Hvidtfeldt 2023; Kayyal-Tarabeia 2024; Poulsen 2023; Prada 2021; Yu 2022 [32,44,50,52,62,63,73]
EDCs (non-pesticide)	8	4	1	3	Mukherjee Das 2022; Omoike 2021; Peng 2023; Tang 2024 [58,59,61,69]
Miscellaneous	8	5	0	3	Garcia-Perez 2018; Carreras 2020; Cazzolla Gatti 2023; Chen 2018; Vieira 2019 [19,34,36,38,70]
Total	51	34	6	11	

## Data Availability

Data sharing is not applicable to this article, as no new data were created or analyzed in this study. All relevant data are contained within the article.

## Abbreviations

The following abbreviations are used in this manuscript:

Abbreviation	Full Term
AOD	Aerosol Optical Depth
BPA	Bisphenol A
BRCA1	Breast Cancer Gene 1
BRCA2	Breast Cancer Gene 2
CDC	Centers for Disease Control and Prevention
DALYs	Disability-Adjusted Life-Years
DCIS	Ductal carcinoma in situ
DDT	Dichlorodiphenyltrichloroethane
DEHP	Di(2-ethylhexyl) phthalate
DNA	Deoxyribonucleic Acid
DOI	Digital object identifier
EC	Elemental Carbon
EDC	Endocrine-Disrupting Chemical
EPA	Environmental Protection Agency
EQI	Environmental Quality Index
GIS	geographic information systems
HER1/2	Human Epidermal Growth Factor Receptor 1/2
IARC	International Agency for Research on Cancer
JBI	Joanna Briggs Institute
LAN	Outdoor light at night
LUR	Land-use regression
MDPI	Multidisciplinary Digital Publishing Institute
NATA	National Air Toxics Assessment
NO_2_	Nitrogen Dioxide
NOx	Nitrogen oxides
PAH	Polycyclic Aromatic Hydrocarbon
PCBs	Polychlorinated biphenyls
PFCs	perfluorinated chemicals
PICOS	Population, Intervention, Comparator, Outcomes, Study Design
PM	Particulate Matter
PM_10_	Fine Particulate Matter 10 μm
PM_2.5_	Fine Particulate Matter 2.5 μm
PRISMA	Preferred Reporting Items for Systematic Reviews and Meta-Analyses
PRTR	Pollutant Release and Transfer Register
PVC	Polyvinyl Chloride
TX	Texas (state abbreviation)
URL	Uniform Resource Locator
USA	United States of America
UV	Ultraviolet
YAP	Yes-Associated Protein

## Appendix A

**Table A1:** Quality Assessment Template, derived from JBI framework.

Domain	Question	Low Risk	High Risk	Unclear
Study Design	What was the study design? (Cohort/Case–control/Cross-sectional/Ecological/Other)	Study design appropriate and clearly reported.	Study design inappropriate for the question or unclear.	Insufficient information.
Population/Sampling	Was the population clearly defined and was sample size adequate?	Population described + sample size adequate/justified.	Poorly defined population or inadequate sample.	Not enough detail to judge.
Exposure Measurement	Was exposure measured validly and reliably, and was geographic/industrial context described?	Validated exposure (registry, monitoring, modelled); geography clear.	Self-report only or unclear exposure source.	Insufficient detail.
Outcome Measurement	Was breast cancer outcome clearly defined and measured appropriately (registry/pathology vs. self-report)? Were subtypes reported?	Registry/pathology data, subtypes and stratifications included.	Self-report or vague/unclear outcomes.	No detail.
Confounding/Comparability	Were key confounders identified and controlled (age, SES, BMI, family history, lifestyle, reproductive factors)?	Most key confounders controlled.	Minimal/no adjustment.	Not reported.
Bias/Data Quality	Were missing data addressed and key biases acknowledged (exposure misclassification, residual confounding)?	Missing data handled + biases discussed.	No handling or acknowledgement.	Not described.
Overall Appraisal	Considering all domains, what is the overall study quality?	Strong across domains.	Serious limitations across multiple domains.	Evidence insufficient.

## References

[1] Arzanova E., Meyrovitz H.N. (2022). The Epidemiology of Breast Cancer. Breast Cancer.

[2] Obeagu E.I., Obeagu G.U. (2024). Breast Cancer: A Review of Risk Factors and Diagnosis. Medicine.

[3] Breast Cancer. https://www.who.int/news-room/fact-sheets/detail/breast-cancer.

[4] Wilkinson L., Gathani T. (2022). Understanding Breast Cancer as a Global Health Concern. Br. J. Radiol..

[5] Colditz G.A., Bohlke K. (2014). Priorities for the Primary Prevention of Breast Cancer. CA Cancer J. Clin..

[6] Kuchenbaecker K.B., Hopper J.L., Barnes D.R., Phillips K.-A., Mooij T.M., Roos-Blom M.-J., Jervis S., Van Leeuwen F.E., Milne R.L., Andrieu N. (2017). Risks of Breast, Ovarian, and Contralateral Breast Cancer for *BRCA1* and *BRCA2* Mutation Carriers. JAMA.

[7] Chang C.-J., Ish J.L., Chang V.C., Daniel M., Jones R.R., White A.J. (2024). Exposure to Per- and Polyfluoroalkyl Substances and Breast Cancer Risk: A Systematic Review and Meta-Analysis of Epidemiologic Studies. Am. J. Epidemiol..

[8] Fenton S.E., Birnbaum L.S. (2015). Timing of Environmental Exposures as a Critical Element in Breast Cancer Risk. J. Clin. Endrocrinol. Metab..

[9] Zeinomar N., Oskar S., Kehm R.D., Sahebzeda S., Terry M.B. (2020). Environmental Exposures and Breast Cancer Risk in the Context of Underlying Susceptibility: A Systematic Review of the Epidemiological Literature. Environ. Res..

[10] Saxena V. (2025). Water Quality, Air Pollution, and Climate Change: Investigating the Environmental Impacts of Industrialization and Urbanization. Water Air Soil Pollut..

[11] Rudel R.A., Fenton S.E., Ackerman J.M., Euling S.Y., Makris S.L. (2011). Environmental Exposures and Mammary Gland Development: State of the Science, Public Health Implications, and Research Recommendations. Environ. Health Perspect..

[12] Brody J.G., Rudel R.A. (2003). Environmental Pollutants and Breast Cancer. Environ. Health Perspect..

[13] CDC Agency for Toxic Substances and Disease Registry. https://www.atsdr.cdc.gov/index.html.

[14] Calaf G.M., Ponce-Cusi R., Aguayo F., Muñoz J.P., Bleak T.C. (2020). Endocrine Disruptors from the Environment Affecting Breast Cancer (Review). Oncol. Lett..

[15] Birnbaum L.S., Fenton S.E. (2003). Cancer and Developmental Exposure to Endocrine Disruptors. Environ. Health Perspect..

[16] Soto A.M., Sonnenschein C. (2010). Environmental Causes of Cancer: Endocrine Disruptors as Carcinogens. Nat. Rev. Endocrinol..

[17] Goldberg M., D’Aloisio A.A., O’Brien K.M., Zhao S., Sandler D.P. (2020). Pubertal Timing and Breast Cancer Risk in the Sister Study Cohort. Breast Cancer Res..

[18] Terry M.B., Michels K.B., Brody J.G., Byrne C., Chen S., Jerry D.J., Malecki K.M.C., Martin M.B., Miller R.L., Neuhausen S.L. (2019). Environmental Exposures during Windows of Susceptibility for Breast Cancer: A Framework for Prevention Research. Breast Cancer Res..

[19] García-Pérez J., Lope V., Pérez-Gómez B., Molina A.J., Tardón A., Díaz Santos M.A., Ardanaz E., O’Callaghan-Gordo C., Altzibar J.M., Gómez-Acebo I. (2018). Risk of Breast Cancer and Residential Proximity to Industrial Installations: New Findings from a Multicase-Control Study (MCC-Spain). Environ. Pollut..

[20] Gasca-Sanchez F.M., Santuario-Facio S.K., Ortiz-López R., Rojas-Martinez A., Mejía-Velázquez G.M., Garza-Perez E.M., Hernández-Hernández J.A., del Carmen López-Sánchez R., Cardona-Huerta S., Santos-Guzman J. (2021). Spatial Interaction between Breast Cancer and Environmental Pollution in the Monterrey Metropolitan Area. Heliyon.

[21] Pan S.Y., Morrison H., Gibbons L., Zhou J., Wen S.W., DesMeules M., Mao Y. (2011). Canadian Cancer Registries Epidemiology Research Group Breast Cancer Risk Associated with Residential Proximity to Industrial Plants in Canada. J. Occup. Environ. Med..

[22] Praud D., Deygas F., Amadou A., Bouilly M., Turati F., Bravi F., Xu T., Grassot L., Coudon T., Fervers B. (2023). Traffic-Related Air Pollution and Breast Cancer Risk: A Systematic Review and Meta-Analysis of Observational Studies. Cancers.

[23] Homaei Shandiz F., Hadizadeh Talasaz Z. (2017). The Relationship between Breast Cancer and Air Pollution: Review Article. Rev. Clin. Med..

[24] White A.J., Weinberg C.R., O’Meara E.S., Sandler D.P., Sprague B.L. (2019). Airborne Metals and Polycyclic Aromatic Hydrocarbons in Relation to Mammographic Breast Density. Breast Cancer Res..

[25] Thong M.S.Y., Mols F., Stein K.D., Smith T., Coebergh J.-W.W., van de Poll-Franse L.V. (2013). Population-Based Cancer Registries for Quality-of-Life Research. Cancer.

[26] List J.M., O’Connor J.M. (2020). How Should Low- and Middle-Income Countries Motivate Equity in Cancer Prevention and Control?. Ethics.

[27] Tolentino-Rodriguez L., Chkeir M., Pofagi V., Ahindu I., Toniolo J., Erazo A., Preux P.-M., Blanquet V., Vergonjeanne M., Parenté A. (2025). Breast Cancer Characteristics in Low- and Middle-Income Countries: An Umbrella Review. Cancer Epidemiol..

[28] Amadou A., Praud D., Coudon T., Danjou A.M.N., Faure E., Leffondré K., Le Romancer M., Severi G., Salizzoni P., Mancini F.R. (2020). Chronic Long-Term Exposure to Cadmium Air Pollution and Breast Cancer Risk in the French E3N Cohort. Int. J. Cancer.

[29] Amadou A., Praud D., Coudon T., Danjou A., Faure E., Deygas F., Grassot L., Leffondré K., Severi G., Salizzoni P. (2021). Exposure to Airborne Cadmium and Breast Cancer Stage, Grade and Histology at Diagnosis: Findings from the E3N Cohort Study. Sci. Rep..

[30] Amadou A., Praud D., Coudon T., Deygas F., Grassot L., Faure E., Couvidat F., Caudeville J., Bessagnet B., Salizzoni P. (2021). Risk of Breast Cancer Associated with Long-Term Exposure to Benzo[a]Pyrene (BaP) Air Pollution: Evidence from the French E3N Cohort Study. Environ. Int..

[31] Amadou A., Praud D., Coudon T., Deygas F., Grassot L., Dubuis M., Faure E., Couvidat F., Caudeville J., Bessagnet B. (2023). Long-Term Exposure to Nitrogen Dioxide Air Pollution and Breast Cancer Risk: A Nested Case-Control within the French E3N Cohort Study. Environ. Pollut..

[32] Arif I., Adams M.D., Johnson M.T.J. (2024). A Meta-Analysis of the Carcinogenic Effects of Particulate Matter and Polycyclic Aromatic Hydrocarbons. Environ. Pollut..

[33] Arrebola J.P., Belhassen H., Artacho-Cordón F., Ghali R., Ghorbel H., Boussen H., Perez-Carrascosa F.M., Expósito J., Hedhili A., Olea N. (2015). Risk of Female Breast Cancer and Serum Concentrations of Organochlorine Pesticides and Polychlorinated Biphenyls: A Case-Control Study in Tunisia. Sci. Total Environ..

[34] Carreras G., Lachi A., Boffi R., Clancy L., Gallus S., Fernández E., López M.J., Soriano J.B., López Nicolás Á., Semple S. (2020). Burden of Disease from Breast Cancer Attributable to Smoking and Second-Hand Smoke Exposure in Europe. Int. J. Cancer.

[35] Carroll R., Ish J., Sandler D., White A., Zhao S. (2023). Understanding the Role of Environmental and Socioeconomic Factors in the Geographic Variation of Breast Cancer Risk in the US-Wide Sister Study. Environ. Res..

[36] Cazzolla Gatti R., Di Paola A., Monaco A., Velichevskaya A., Amoroso N., Bellotti R. (2023). The Spatial Association between Environmental Pollution and Long-Term Cancer Mortality in Italy. Sci. Total Environ..

[37] Cazzolla Gatti R. (2021). Why We Will Continue to Lose Our Battle with Cancers If We Do Not Stop Their Triggers from Environmental Pollution. Int. J. Environ. Res. Public Health.

[38] Chen X. (2018). A Temporal Analysis of the Association between Breast Cancer and Socioeconomic and Environmental Factors. GeoJournal.

[39] Cohen G., Levy I., Yuval, Kark J.D., Levin N., Witberg G., Iakobishvili Z., Bental T., Broday D.M., Steinberg D.M. (2018). Chronic Exposure to Traffic-Related Air Pollution and Cancer Incidence among 10,000 Patients Undergoing Percutaneous Coronary Interventions: A Historical Prospective Study. Eur. J. Prev. Cardiol..

[40] Coudon T., Praud D., Faure E., Danjou A., Mancini F., Severi G., Fervers B. (2020). GEO3N: Environmental Exposure to Dioxins and Breast Cancer Risk in the E3N Cohort. Environ. Risues Sante.

[41] Da Silva M., Moreno M., De Sá C., Rizzi C., Ribeiro E., Ripke M., Corralo V. (2024). Mortality from Breast Cancer and Use of Pesticides in the Western Mesoregion of Santa Catarina—Brazil. Rev. Bras. Cienc. Ambient..

[42] Danjou A., Coudon T., Praud D., Lévêque E., Faure E., Salizzoni P., Le Romancer M., Severi G., Mancini F., Leffondré K. (2019). Long-Term Airborne Dioxin Exposure and Breast Cancer Risk in a Case-Control Study Nested within the French E3N Prospective Cohort. Environ. Int..

[43] Deygas F., Amadou A., Coudon T., Grassot L., Couvidat F., Bessagnet B., Faure E., Salizzoni P., Gulliver J., Caudeville J. (2021). Long-Term Atmospheric Exposure to PCB153 and Breast Cancer Risk in a Case-Control Study Nested in the French E3N Cohort from 1990 to 2011. Environ. Res..

[44] Duboeuf M., Amadou A., Coudon T., Grassot L., Ramel-Delobel M., Faure E., Salizzoni P., Gulliver J., Severi G., Mancini F.R. (2024). Long-Term Exposure to Air Pollution at Residential and Workplace Addresses and Breast Cancer Risk: A Case-Control Study Nested in the French E3N-Générations Cohort from 1990 to 2011. Eur. J. Cancer.

[45] DuPre N.C., Hart J.E., Holmes M.D., Poole E.M., James P., Kraft P., Laden F., Tamimi R.M. (2019). Particulate Matter and Traffic-Related Exposures in Relation to Breast Cancer Survival. Cancer. Epidemiol. Biomark. Prev..

[46] Garcia E., Hurley S., Nelson D., Hertz A., Reynolds P. (2015). Hazardous Air Pollutants and Breast Cancer Risk in California Teachers: A Cohort Study. Environ. Health.

[47] García-Pérez J., Pérez-Abad N., Lope V., Castelló A., Pollán M., González-Sánchez M., Valencia J.L., López-Abente G., Fernández-Navarro P. (2016). Breast and Prostate Cancer Mortality and Industrial Pollution. Environ. Pollut..

[48] Gearhart-Serna L.M., Hoffman K., Devi G.R. (2020). Environmental Quality and Invasive Breast Cancer. Cancer Epidemiol. Biomark. Prev..

[49] He Y., Wang X., Wu K. (2016). Evaluating Breast Cancer Risk under Exposure to Environmental Estrogen-like Chemicals. Pol. J. Environ. Stud..

[50] Hvidtfeldt U.A., Chen J., Rodopoulou S., Strak M., de Hoogh K., Andersen Z.J., Bellander T., Brandt J., Fecht D., Forastiere F. (2023). Breast Cancer Incidence in Relation to Long-Term Low-Level Exposure to Air Pollution in the ELAPSE Pooled Cohort. Cancer Epidemiol. Biomark. Prev..

[51] Ilozumba M.N., Shelver W.L., Hong C.-C., Ambrosone C.B., Cheng T.-Y.D. (2022). Urinary Concentrations of Triclosan, Bisphenol A, and Brominated Flame Retardants and the Association of Triclosan with Demographic Characteristics and Body Fatness among Women with Newly Diagnosed Breast Cancer. Int. J. Environ. Res. Public Health.

[52] Kayyal-Tarabeia I., Zick A., Kloog I., Levy I., Blank M., Agay-Shay K. (2024). Beyond Lung Cancer: Air Pollution and Bladder, Breast and Prostate Cancer Incidence. Int. J. Epidemiol..

[53] Large C., Wei Y. (2017). Geographic Variations in Female Breast Cancer Incidence in Relation to Ambient Air Emissions of Polycyclic Aromatic Hydrocarbons. Environ. Sci. Pollut. Res..

[54] Le Provost B., Parent M.-É., Villeneuve P.J., Waddingham C.M., Brook J.R., Lavigne E., Dugandzic R., Harris S.A. (2024). Residential Exposure to Ambient Fine Particulate Matter (PM_2.5_) and Nitrogen Dioxide (NO_2_) and Incident Breast Cancer among Young Women in Ontario, Canada. Cancer Epidemiol..

[55] Liu G., Cai W., Liu H., Jiang H., Bi Y., Wang H. (2021). The Association of Bisphenol a and Phthalates with Risk of Breast Cancer: A Meta-Analysis. Int. J. Environ. Res. Public Health.

[56] Mekonen S., Ibrahim M., Astatkie H., Abreha A. (2021). Exposure to Organochlorine Pesticides as a Predictor to Breast Cancer: A Case-Control Study among Ethiopian Women. PLoS ONE.

[57] Michel-Ramirez G., Recio-Vega R., Lantz R.C., Gandolfi A.J., Olivas-Calderon E., Chau B.T., Amistadi M.K. (2020). Assessment of YAP Gene Polymorphisms and Arsenic Interaction in Mexican Women with Breast Cancer. J. Appl. Toxicol..

[58] Mukherjee Das A., Gogia A., Garg M., Elaiyaraja A., Arambam P., Mathur S., Babu-Rajendran R., Deo S.V.S., Kumar L., Das B.C. (2022). Urinary Concentration of Endocrine-Disrupting Phthalates and Breast Cancer Risk in Indian Women: A Case-Control Study with a Focus on Mutations in Phthalate-Responsive Genes. Cancer Epidemiol..

[59] Omoike O.E., Pack R.P., Mamudu H.M., Liu Y., Wang L. (2021). A Cross-Sectional Study of the Association between Perfluorinated Chemical Exposure and Cancers Related to Deregulation of Estrogen Receptors. Environ. Res..

[60] Panis C., Candiotto L.Z.P., Gaboardi S.C., Gurzenda S., Cruz J., Castro M., Lemos B. (2022). Widespread Pesticide Contamination of Drinking Water and Impact on Cancer Risk in Brazil. Environ. Int..

[61] Peng S., Dong S., Gong C., Chen X., Du H., Zhan Y., Yang Z. (2023). Evidence-Based Identification of Breast Cancer and Associated Ovarian and Uterus Cancer Risk Components in Source Waters from High Incidence Area in the Pearl River Basin, China. Sci. Total Environ..

[62] Poulsen A.H., Hvidtfeldt U.A., Sørensen M., Pedersen J.E., Ketzel M., Brandt J., Geels C., Christensen J.H., Raaschou-Nielsen O. (2023). Air Pollution with NO_2_, PM_2.5_, and Elemental Carbon in Relation to Risk of Breast Cancer– a Nationwide Case-Control Study from Denmark. Environ. Res..

[63] Prada D., Baccarelli A.A., Terry M.B., Valdéz L., Cabrera P., Just A., Kloog I., Caro H., García-Cuellar C., Sánchez-Pérez Y. (2021). Long-Term PM_2.5_ Exposure before Diagnosis Is Associated with Worse Outcome in Breast Cancer. Breast Cancer Res. Treat..

[64] Praud D., Amadou A., Coudon T., Duboeuf M., Mercoeur B., Faure E., Grassot L., Danjou A.M., Salizzoni P., Couvidat F. (2025). Association between Chronic Long-Term Exposure to Airborne Dioxins and Breast Cancer. Int. J. Hyg. Environ. Health.

[65] Shekarrizfard M., Valois M.-F., Goldberg M.S., Crouse D., Ross N., Parent M.-E., Yasmin S., Hatzopoulou M. (2015). Investigating the Role of Transportation Models in Epidemiologic Studies of Traffic Related Air Pollution and Health Effects. Environ. Res..

[66] Shekarrizfard M., Valois M., Weichenthal S., Goldberg M., Fallah-Shorshani M., Cavellin L., Crouse D., Parent M., Hatzopoulou M. (2018). Investigating the Effects of Multiple Exposure Measures to Traffic-Related Air Pollution on the Risk of Breast and Prostate Cancer. J. Transp. Health.

[67] Silva A.M.C., Campos P.H.N., Mattos I.E., Hajat S., Lacerda E.M., Ferreira M.J.M. (2019). Environmental Exposure to Pesticides and Breast Cancer in a Region of Intensive Agribusiness Activity in Brazil: A Case-Control Study. Int. J. Environ. Res. Public Health.

[68] Stults W.P., Wei Y. (2018). Ambient Air Emissions of Polycyclic Aromatic Hydrocarbons and Female Breast Cancer Incidence in US. Med. Oncol..

[69] Tang L., Wang Y., Yan W., Zhang Z., Luo S., Wen Q., Wang S., Zhou N., Chen Q., Xu Y. (2024). Exposure to Di-2-Ethylhexyl Phthalate and Breast Neoplasm Incidence: A Cohort Study. Sci. Total Environ..

[70] Vieira V.M., Vopham T., Bertrand K.A., James P., Dupré N., Tamimi R.M., Laden F., Hart J.E. (2019). Contribution of Socioeconomic and Environmental Factors to Geographic Disparities in Breast Cancer Risk in the Nurses’ Health Study II. Environ. Epidemiol..

[71] Waddingham C.M., Hinton P., Villeneuve P.J., Brook J.R., Lavigne E., Larsen K., King W.D., Wen D., Meng J., Zhang J. (2024). Exposure to Ambient Polycyclic Aromatic Hydrocarbons and Early-Onset Female Breast Cancer in a Case-Control Study in Ontario, Canada. Environ. Epidemiol..

[72] Yaghjyan L., Arao R., Brokamp C., O’Meara E.S., Sprague B.L., Ghita G., Ryan P. (2017). Association between Air Pollution and Mammographic Breast Density in the Breast Cancer Surveilance Consortium. Breast Cancer Res..

[73] Yu P., Xu R., Li S., Coelho M.S.Z.S., Saldiva P.H.N., Sim M.R., Abramson M.J., Guo Y. (2022). Associations between Long-Term Exposure to PM_2.5_ and Site-Specific Cancer Mortality: A Nationwide Study in Brazil between 2010 and 2018. Environ. Pollut..

[74] Zhai Y., Amadou A., Mercier C., Praud D., Faure E., Iwaz J., Severi G., Mancini F., Coudon T., Fervers B. (2022). The Impact of Left Truncation of Exposure in Environmental Case-Control Studies: Evidence from Breast Cancer Risk Associated with Airborne Dioxin. Eur. J. Epidemiol..

[75] White A.J., O’Brien K.M., Niehoff N.M., Carroll R., Sandler D.P. (2019). Metallic Air Pollutants and Breast Cancer Risk in a Nationwide Cohort Study. Epidemiology.

[76] Northeim K., Tiwari C., Oppong J. (2021). Surface Ozone Monitoring and Policy: A Geospatial Decision Support Tool for Suitable Location of Monitoring Stations in Urban Areas. Environ. Sci. Policy.

[77] Northeim K., Oppong J.R., Northeim K., Oppong J.R. (2023). Mapping Health Fragility and Vulnerability in Air Pollution–Monitoring Networks in Dallas–Fort Worth. Int. J. Environ. Res. Public Health.

[78] Tarhonska K., Lesicka M., Janasik B., Roszak J., Reszka E., Braun M., Kołacińska-Wow A., Jabłońska E. (2022). Cadmium and Breast Cancer—Current State and Research Gaps in the Underlying Mechanisms. Toxicol. Lett..

[79] Reich B.J., Yang S., Guan Y., Giffin A.B., Miller M.J., Rappold A. (2021). A Review of Spatial Causal Inference Methods for Environmental and Epidemiological Applications. Int. Stat. Rev..

[80] Shaw P.A., Deffner V., Keogh R.H., Tooze J.A., Dodd K.W., Küchenhoff H., Kipnis V., Freedman L.S. (2018). Epidemiologic Analyses with Error-Prone Exposures: Review of Current Practice and Recommendations. Ann. Epidemiol..

[81] Mansournia M.A., Poole C. (2023). Case-Control Matching on Confounders Revisited. Eur. J. Epidemiol..

[82] Zhang L., Mukherjee B., Ghosh M., Gruber S., Moreno V. (2008). Accounting for Error Due to Misclassification of Exposures in Case-Control Studies of Gene-Environment Interaction. Stat. Med..

[83] Chen C., Chen H., Kaufman J.S., Benmarhnia T. (2024). Differential Participation, a Potential Cause of Spurious Associations in Observational Cohorts in Environmental Epidemiology. Epidemiology.

[84] Rocha P.R.S., Oliveira V.D., Vasques C.I., Dos Reis P.E.D., Amato A.A. (2021). Exposure to Endocrine Disruptors and Risk of Breast Cancer: A Systematic Review. Crit. Rev. Oncol. Hematol..

[85] Dominici F., Zanobetti A., Schwartz J., Braun D., Sabath B., Wu X. (2022). Assessing Adverse Health Effects of Long-Term Exposure to Low Levels of Ambient Air Pollution: Implementation of Causal Inference Methods. Res. Rep. Health Eff. Inst..

[86] Hoek G., Vienneau D., de Hoogh K. (2024). Does Residential Address-Based Exposure Assessment for Outdoor Air Pollution Lead to Bias in Epidemiological Studies?. Environ. Health.

[87] Webster T.F. (2007). Bias Magnification in Ecologic Studies: A Methodological Investigation. Environ. Health.

[88] Shih Y.-C.T., Bradley C., Yabroff K.R. (2023). Ecological and Individualistic Fallacies in Health Disparities Research. J. Natl. Cancer Inst..

[89] Wacholder S., McLaughlin J.K., Silverman D.T., Mandel J.S. (1992). Selection of Controls in Case-Control Studies. I. Principles. Am. J. Epidemiol..

[90] Czaczkowska L., Jabłońska E., Ratajczak-Wrona W. (2025). Endocrine Disruptors and Breast Cancer: A Comprehensive Review. Biomedicines.

[91] Day D.B., Sathyanarayana S., LeWinn K.Z., Karr C.J., Mason W.A., Szpiro A.A. (2022). A Permutation Test-Based Approach to Strengthening Inference on the Effects of Environmental Mixtures: Comparison between Single-Index Analytic Methods. Environ. Health Perspect..

[92] Ma X., Zou B., Deng J., Gao J., Longley I., Xiao S., Guo B., Wu Y., Xu T., Xu X. (2024). A Comprehensive Review of the Development of Land Use Regression Approaches for Modeling Spatiotemporal Variations of Ambient Air Pollution: A Perspective from 2011 to 2023. Environ. Int..

[93] Rudel R.A., Ackerman J.M., Attfield K.R., Brody J.G. (2014). New Exposure Biomarkers as Tools for Breast Cancer Epidemiology, Biomonitoring, and Prevention: A Systematic Approach Based on Animal Evidence. Environ. Health Perspect..

[94] Salama Y., Chennaoui M., Salama Y., Chennaoui M. (2024). A Critical Review of Biomarkers in Toxicology and Risk Assessment of Environmental Pollutants. Ann. Environ. Sci. Toxicol..

[95] Aroma R J., Raimond K. (2016). An Overview of Technological Revolution in Satellite Image Analysis. J. Eng. Sci. Technol. Rev..

[96] Diao M., Holloway T., Choi S., O’Neill S.M., Al-Hamdan M.Z., van Donkelaar A., Martin R.V., Jin X., Fiore A.M., Henze D.K. (2019). Methods, Availability, and Applications of PM_2.5_ Exposure Estimates Derived from Ground Measurements, Satellite, and Atmospheric Models. J. Air Waste Manag. Assoc..

[97] Orozco-Acosta E., Adin A., Ugarte M.D. (2023). Big Problems in Spatio-Temporal Disease Mapping: Methods and Software. Comput. Methods Programs Biomed..

[98] Tim U.S. (1995). The Application of GIS in Environmental Health Sciences: Opportunities and Limitations. Environ. Res..

[99] Kehm R.D., Lloyd S.E., Burke K.R., Terry M.B. (2025). Advancing Environmental Epidemiologic Methods to Confront the Cancer Burden. Am. J. Epidemiol..

[100] Fairbrother M. (2013). Rich People, Poor People, and Environmental Concern: Evidence across Nations and Time. Eur. Sociol. Rev..

[101] Koval L.E., Dionisio K.L., Friedman K.P., Isaacs K.K., Rager J.E. (2022). Environmental Mixtures and Breast Cancer: Identifying Co-Exposure Patterns between Understudied vs Breast Cancer-Associated Chemicals Using Chemical Inventory Informatics. J. Expo. Sci. Environ. Epidemiol..

[102] Stordal B., Harvie M., Antoniou M.N., Bellingham M., Chan D.S.M., Darbre P., Karlsson O., Kortenkamp A., Magee P., Mandriota S. (2024). Breast Cancer Risk and Prevention in 2024: An Overview from the Breast Cancer UK—Breast Cancer Prevention Conference. Cancer Med..

[103] Zahnd W.E., McLafferty S.L., Eberth J.M. (2019). Multilevel Analysis in Rural Cancer Control: A Conceptual Framework and Methodological Implications. Prev. Med..

[104] Davis D.L., Axelrod D., Bailey L., Gaynor M., Sasco A.J. (1998). Rethinking Breast Cancer Risk and the Environment: The Case for the Precautionary Principle. Environ. Health Perspect..

[105] Brody J.G., Tickner J., Rudel R.A. (2005). Community-Initiated Breast Cancer and Environment Studies and the Precautionary Principle. Environ. Health Perspect..

[106] Doherty B.T., McRitchie S.L., Pathmasiri W.W., Stewart D.A., Kirchner D., Anderson K.A., Gui J., Madan J.C., Hoen A.G., Sumner S.J. (2022). Chemical Exposures Assessed via Silicone Wristbands and Endogenous Plasma Metabolomics during Pregnancy. J. Expo. Sci. Environ. Epidemiol..

[107] Martenies S.E., Zhang M., Corrigan A.E., Kvit A., Shields T., Wheaton W., Him D.A., Aschner J., Talavera-Barber M.M., Barrett E.S. (2023). Developing a National-Scale Exposure Index for Combined Environmental Hazards and Social Stressors and Applications to the Environmental Influences on Child Health Outcomes (ECHO) Cohort. Int. J. Environ. Res. Public Health.

[108] Santaliz Casiano A., Lee A., Teteh D., Madak Erdogan Z., Treviño L. (2022). Endocrine-Disrupting Chemicals and Breast Cancer: Disparities in Exposure and Importance of Research Inclusivity. Endocrinology.

[109] European Commission Zero Pollution Action Plan—Environment. https://environment.ec.europa.eu/strategy/zero-pollution-action-plan_en.

[110] Environmental Inequalities. https://www.eea.europa.eu/en/topics/in-depth/environmental-inequalities.

